# Synergistic Design of Multicomponent Carbon Fillers and Structures for Emi Shielding Rubbers

**DOI:** 10.1002/smsc.202500410

**Published:** 2025-10-21

**Authors:** Weijian Zhang, Lechun Deng, Tengxun Yang, Fa Luo, Shifeng Wen, Yun Tang, Hongjing Wu, Qiang Chen

**Affiliations:** ^1^ State Key Laboratory of Solidification Processing School of Materials Science and Engineering Northwestern Polytechnical University Xi'an 710072 China; ^2^ School of Materials Science & Engineering Xi'an University of Architecture & Technology Xi'an Shaanxi 710055 China; ^3^ MOE Key Laboratory of Material Physics and Chemistry under Extraordinary, School of Physics Science and Technology Northwestern Polytechnical University Xi'an 710072 China

**Keywords:** carbon-based conductive rubber composites, composite structural design, electromagnetic interference shielding, filler synergistic effect

## Abstract

With the rapid development of highly integrated electronic devices, electromagnetic interference leakage through assembly gaps has become a critical challenge. Conductive rubber, combining electrical conductivity and elastic compressibility, is widely recognized as a core material for achieving electromagnetic compatibility. Carbon‐based conductive rubbers are attractive for their lightweight and corrosion resistance, but they face the critical bottleneck of achieving high shielding efficiency at low filler loadings. To address this issue, research has shifted from single‐component carbon fillers toward multicomponent synergistic systems and structural designs. This review systematically classifies synergistic systems into carbon–carbon, carbon–metal, and carbon–magnetic types, highlighting their conductive network architectures, shielding mechanisms, and performance trade‐offs. It further emphasizes the coupled optimization between filler systems and rubber structures, which enables significant improvements in shielding effectiveness. Finally, the review outlines future directions, including service reliability, integrated structural–functional design, intelligent responsive materials, and multifunctional sustainable development, providing guidance for the advancement of high‐performance carbon‐based conductive rubbers.

## Graphical abstract

This review systematically discusses synergistic carbon‐based conductive rubbers, emphasizing carbon–carbon, carbon–metal, and carbon–magnetic systems. It highlights coupled optimization between filler networks and rubber structures for high electromagnetic interference shielding efficiency at low loadings, while also analyzing performance trade‐offs, structural design strategies, and outlining perspectives on intelligent responsiveness, service reliability, multifunctional integration, and sustainable development.

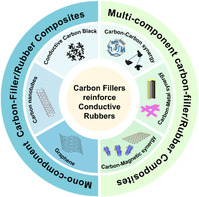

## 1 Introduction

The rapid development of electronic information technology has not only driven social progress and improved quality of life but has also accelerated the evolution of electronic devices toward higher integration, portability, and power. This trend has significantly intensified the issue of electromagnetic interference (EMI),^[^ ^1^, ^2^, ^3^, ^4^, ^5^ ^]^ which can disrupt the normal operation of electronic equipment and may pose potential health risks to humans. Therefore, the development of high‐performance electromagnetic functional materials, particularly efficient EMI shielding materials, has become an urgent necessity.^[^ ^6^, ^7^, ^8^, ^9^, ^10^, ^11^, ^12^ ^]^

Copper, aluminum, and stainless steel are commonly employed in fabricating equipment housings or covers for EMI. However, the inevitable assembly gaps between the housing and cover become the main pathway for electromagnetic leakage and also provide a channel for external EMI to intrude. In addition, the high density, susceptibility to corrosion, and difficulty in processing and forming of metal materials limit their application in many scenarios.^[^ ^13^, ^14^, ^15^, ^16^ ^]^ Compared with other polymer matrices, conductive rubber, which combines electrical conductivity with unique elasticity, compressibility, and sealing ability, is a key material for electromagnetic sealing, making it particularly suitable for EMI gaskets and seals that demand intimate contact under pressure. Among various types of conductive rubber, carbon‐filled conductive rubber has shown broad application prospects due to its advantages of being lightweight, easy to process, and corrosion‐resistant.^[^ ^17^, ^18^, ^19^, ^20^ ^]^

In particular, nanocarbon fillers such as carbon black (CB), carbon nanotubes (CNTs), and graphene possess high aspect ratios, large surface areas, and excellent intrinsic conductivity. These features enable the formation of multiscale conductive networks at relatively low loadings, which improves EMI shielding effectiveness (SE) while preserving the lightweight and flexible characteristics of rubber matrices. In addition, nanofillers can act as reinforcing agents, enhancing the mechanical strength and durability of rubber composites.

Nevertheless, the electromagnetic shielding performance of carbon‐based conductive rubber still falls short compared to that of traditional metal materials. Achieving high shielding efficiency with low filler content therefore remains a core challenge. Although the optimization of single‐component carbon fillers is still a fundamental strategy, the growing demand for advanced electromagnetic protection has shifted attention toward multicomponent synergistic systems. In particular, combining filler synergy with structural strategies (e.g., foams, multilayers, segregated networks) has become a key approach to overcome current limitations, and this dual focus constitutes the central theme of this review.

Given the relative scarcity of systematic reviews on the research progress of rubber‐based EMI shielding composite materials, and the lack of a systematic overview of the synergistic mechanisms of multicomponent fillers and their coupling with structural design, this review systematically combs through the research progress of carbon‐based conductive rubber in the design of efficient filler systems, filler distribution strategies, and structural design of composite materials. It also looks forward to its application prospects and future development directions, with the aim of providing references for the design and development of high‐performance carbon‐based conductive rubber.

## 2 Mechanism of EMI Shielding

The ability of a shielding material to block electromagnetic radiation is quantified using EMI SE, expressed in decibels (dB). The definition of SE is determined by comparing the changes in electromagnetic field strength (or power) at a specific spatial location with and without the presence of the shielding body. The formula for calculating SE is as follows

(1) $$\begin{array}{c} \text{SE}=20\mathrm{lg}(\frac{E_{1}}{E_{2}})=20\text{lg}(\frac{H_{1}}{H_{2}})=10\text{lg}(\frac{P_{1}}{P_{2}} \end{array})$$

In Equation (1), $E_{1}$, $H_{1}$, and $P_{1}$ represent the electric field, magnetic field, and power density in the absence of a shielding body, respectively, while $E_{2}$, $H_{2}$, and $P_{2}$ denote the electric field, magnetic field, and power density in the presence of a shielding body, respectively.

The method for calculating the electromagnetic SE of homogeneous shielding materials is based on Schelkunoff theory.^[^ ^21^, ^22^ ^]^ This theory is grounded in transmission line theory,^[^ ^23^, ^24^ ^]^ which treats the shielding material as a segment of a transmission line. When electromagnetic waves impinge on the outer surface of the shielding material, a portion of the waves is reflected due to impedance mismatch, while the remaining portion penetrates into the shield. During transmission, the waves are attenuated by the shielding material and undergo multiple reflections and transmissions within the shield. According to this theory, the EMI SE of the shielding material can be expressed as

(2) $$\begin{array}{c} {\text{SE}}_{T}={\text{SE}}_{A}+{\text{SE}}_{R}+{\text{SE}}_{M}\left(\text{dB}\right) \end{array}$$

In Equations (2), ${\text{SE}}_{T},\;{\text{SE}}_{A},\;{\text{SE}}_{R},\text{and}\;{\text{SE}}_{M}$ represent the total attenuation effect of the shielding material on electromagnetic waves, the absorption loss of the shielding material, the single‐reflection loss at the surface of the shield, and the multiple reflection loss within the shield, respectively. Furthermore, ${\text{SE}}_{A}$, ${\text{SE}}_{R}$, and ${\text{SE}}_{M}$ can be expressed by Equation ((3), (4))–(5).

(3) $$\begin{array}{c} {\text{SE}}_{A}=131.43t\sqrt{f\mu_{r}\sigma_{r}} \end{array}$$

(4) $$\begin{array}{c} {\text{SE}}_{R}=168.2+10\mathrm{lg}\left(\frac{\sigma_{r}}{f\mu_{r}}\right) \end{array}$$

(5) $$\begin{array}{c} {\text{SE}}_{M}=20\mathrm{lg}\left(1-10^{\frac{\text{SEA}}{10}}\right) \end{array}$$

where, *f* represents the frequency of electromagnetic wave (Hz), *t* represents the thickness of the sample (m), $\mu_{r}$ represents relative permeability, and $\sigma_{r}$ relative conductivity (relative to copper). For thicker materials, when the electromagnetic wave reaches the second boundary of the shielding material, ${\text{SE}}_{M}$ can be neglected. It can be seen from Equation (5) that ${\text{SE}}_{M}$ is related to ${\text{SE}}_{A}$. When the ${\text{SE}}_{A}$.of the shielding material is ≥15 dB, ${\text{SE}}_{M}$ can be neglected.

In summary, reflection, absorption, and multiple reflections remain the classical EMI shielding mechanisms, but their implementation in rubber‐based composites presents both challenges and opportunities. Excessive reflection from highly conductive fillers can lead to secondary interference, making absorption via interfacial polarization or magnetic loss particularly important. Meanwhile, the flexibility and easy processability of rubber enable the design of foams, multilayers, and segregated structures that enhance multiple scattering and impedance matching. Combining these structural advantages with synergistic fillers offers new pathways for advanced EMI shielding design.

## 3 Single‐Component Carbon Fillers

Single‐component filler systems, such as CB, graphene, and CNTs, form a vital foundation for the development of conductive rubber. They offer two key advantages: 1) a simple composition that promotes excellent process compatibility, and 2) a clear relationship between filler loading and composite performance, enabling precise control during industrial manufacturing.

### 3.1 Conductive Carbon Black

Conductive CB is formed by the incomplete combustion or thermal decomposition of hydrocarbon compounds, and is composed of aggregated primary nanoparticles. Its electrical conductivity ranges from 0.1 to 100 S cm^−1^.^[^ ^25^, ^26^ ^]^ It is the most cost‐effective EMI shielding filler in rubber composites. Compared with CNTs or graphene, the inherent electrical conductivity of CB is relatively low. Generally, a loading amount of more than 15 wt% is required to achieve effective EMI shielding.^[^ ^27^ ^]^ However, excessive loading tends to lead to a decrease in the mechanical properties of the composite materials. For example, in chlorinated polyethylene (CPE) rubber, when the loading of conductive CB is increased from 15 to 30 wt%, the EMI SE of the CPE/CB composite material increases from 21.2 to 38.4 dB, while the tensile strength decreases from 23 to 11.1 MPa.^[^ ^28^ ^]^

### 3.2 Graphene

While CB is cost‐effective and widely used, its low intrinsic conductivity limits its effectiveness in demanding EMI shielding environments. In contrast, graphene, a 2D material composed of a single layer of sp^2^‐hybridized carbon atoms, has emerged as a promising filler for enhancing the electromagnetic shielding performance of rubber composites due to its excellent conductivity, stability, and high compatibility.^[^ ^29^, ^30^, ^31^ ^]^ In addition, the compressibility of rubber allows graphene‐based conductive networks to be maintained or even reconstructed under deformation, ensuring stable shielding performance in dynamic conditions, while the layered graphene sheets can occupy microgaps in the matrix to further improve sealing capability along with providing efficient electrical pathways.

Due to the large specific surface area of graphene, the strong interlayer interactions, and the high viscosity of the rubber matrix, it is difficult to disperse graphene in the rubber matrix. Usually, graphene nanoplatelets (GNP) or reduced graphene oxide (RGO) are selected to prepare graphene/rubber conductive composites. GNP are mainly obtained directly from graphite through physical methods such as liquid‐phase exfoliation and mechanical exfoliation. RGO needs to be prepared first by chemically oxidizing graphite to obtain GO, and then the oxygen‐containing functional groups are removed through a reduction process. Due to the presence of a large number of sp^3^ carbon regions, structural defects, and residual oxygen‐containing groups, which seriously hinder the migration of electrons, the electrical conductivity of RGO is usually significantly lower than that of GNP.

#### 3.2.1 Homogeneous Rubber

Homogeneous graphene/rubber composites are typically prepared by mechanical blending followed by hot‐pressing, or by solution (latex) blending with subsequent solvent removal. The latter reduces matrix viscosity and aids dispersion. For example, two‐roll milling of GNP into silicone rubber (SR) yielded an EMI SE of 43.2 dB at 7 wt% GNP in the frequency range of 1 MHz–1 GHz.^[^ ^32^ ^]^ Incorporating GNP into nitrile rubber (NBR) gave 38–77 dB at 4.0 wt% in the frequency range of 1–12 GHz.^[^ ^33^ ^]^ To further improve RGO dispersion, Das et al.^[^ ^34^ ^]^ used a solution‐then‐mechanical route in which carboxylated nitrile butadiene rubber (XNBR) was dissolved in tetrahydrofuran, RGO was dispersed, and the solvent removed, followed by mechanical mixing of other additives; the resulting composite with 15 phr RGO achieved 34 dB in the X‐band.

#### 3.2.2 Framework Rubber

**Figure 1:**
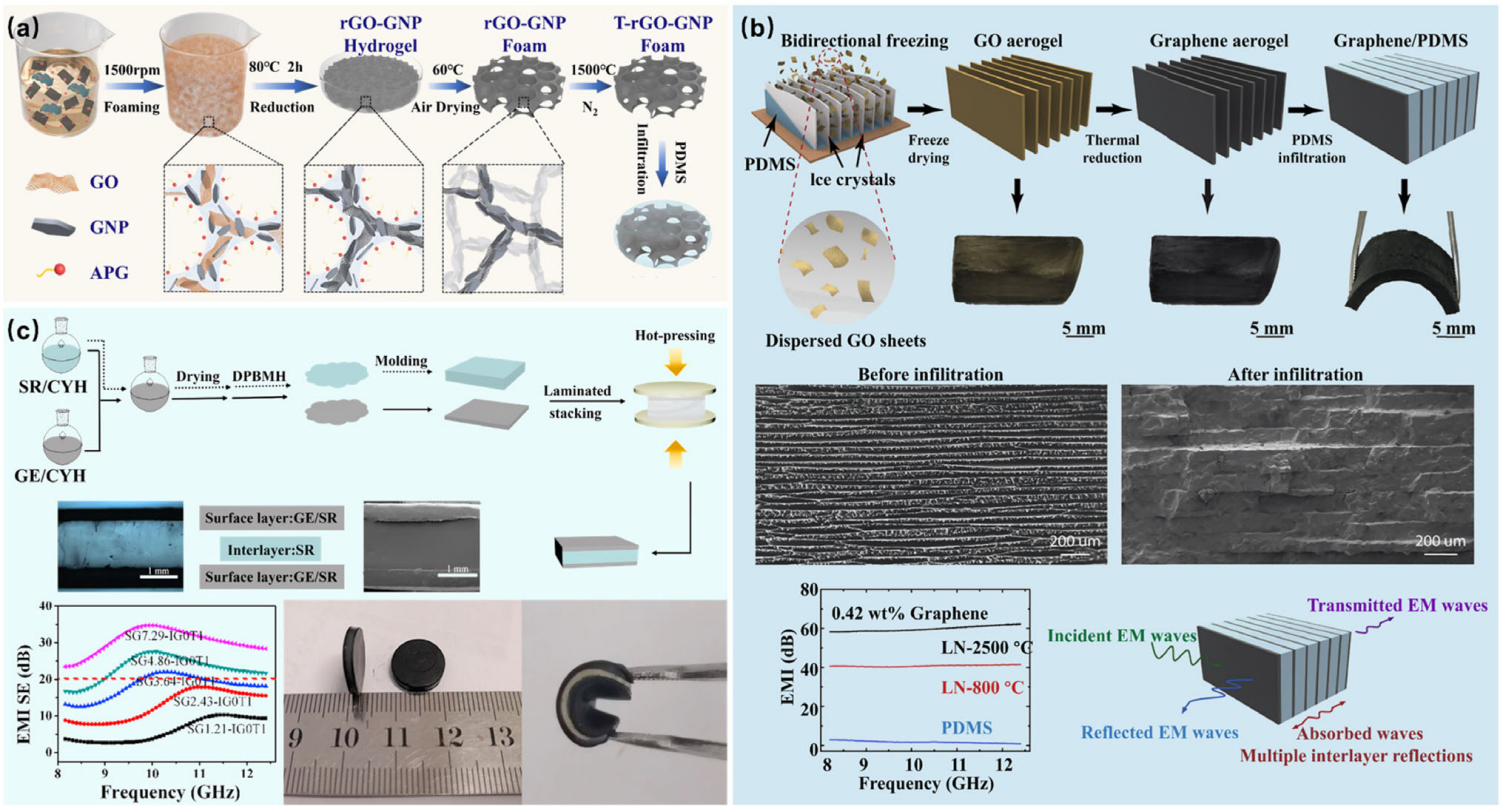
Fabrication schematics of a) template‐assisted GNP/PDMS conductive foam, b) bidirectional‐freezing and high‐temperature annealing for oriented graphene/PDMS composites, and c) sandwich‐structured RGO/SR composites.^[^ ^35^, ^36^, ^37^ ^]^

In addition to homogeneous structures, constructing a conductive framework is an important strategy for controlling the distribution of graphene within the rubber matrix, effectively utilizing fillers, and significantly improving EMI shielding performance. The conductive framework enhances the composite's conductivity by providing continuous conductive pathways, thereby increasing electromagnetic wave attenuation. For example, Li et al.^[^ ^35^ ^]^ used surfactant‐induced bubbles as templates to prepare a 3D conductive framework of reduced RGO and GNP through high‐temperature annealing, as shown in **Figure** **1**a. After encapsulating with polydimethylsiloxane (PDMS), the resulting composite material exhibited an EMI SE of ≈90 dB, with a graphene content of only 18.1 wt%. Furthermore, controlling the orientation of graphene within the matrix can also create conductive frameworks with specific structures. Gao et al.^[^ ^36^ ^]^ prepared oriented layered graphene aerogels using a bidirectional freezing technique and reduced them in a hydrogen/argon atmosphere at 800 °C, followed by encapsulation with PDMS, as shown in Figure 1b. When the graphene content was 0.42 wt%, the EMI SE of the composite material was 42 dB. After annealing at 2500 °C, the EMI SE of the graphene aerogel increased to about 65 dB, reaching a level comparable to that of copper foil.

#### 3.2.3 Multilayer Rubber

In addition to constructing a conductive framework, it is also possible to design multilayer conductive rubber. This structure enhances the attenuation of electromagnetic waves by promoting their multiple reflections, scattering, and absorption between different layers. A common structural design for rubber is the construction of multilayer or sandwich structures. For instance, Wang et al.^[^ ^37^ ^]^ prepared RGO/SR composites using a solution blending method and assembled them into a sandwich structure, as shown in Figure 1c. The results showed that within the X‐band frequency range, the EMI SE of the uniform structure containing 3 wt% RGO was less than 20 dB at a thickness of 1 mm. In contrast, the EMI SE of the sandwich structure composite was significantly enhanced, reaching 30.42 dB. This improvement is attributed to the multiple coherent reflections of electromagnetic waves between the two parallel reflective planes, leading to the phenomenon of Fabry–Pérot cavity resonance. For clarity, **Table** **1** provides a comprehensive comparison of the electromagnetic shielding performance of different graphene/rubber composites.

**Table 1:** Comparison of electromagnetic shielding performance of different graphene/rubber composites.

Matrix	Filler loading of graphene	Thickness [mm]	EMI SE [dB]	Frequency [GHz]	Refs.
SR	7 wt% GNP	1	43.2	0.001–1	[32]
NBR	4 wt% GNP	2	77	1–12	[33]
XNBR	15 phr RGO	1	34	8–12	[34]
PDMS	18.1 wt% GNP	2	86	8–12	[35]
PDMS	0.42 wt% GNP	4	65	8–12	[36]
SR	3 wt% RGO	1.7	34.7	8–12	[37]

### 3.3 CNTs

CNTs are seamless cylindrical structures formed by rolling single or multiple layers of graphene sheets at a specific helical angle. Depending on the rolling method, CNTs can be classified into single‐walled CNTs and multiwalled CNTs, with intrinsic electrical conductivities typically ranging from 10^4^ to 10^6^ S m^−1^. Due to their 1D structure, CNTs can effectively form conductive networks in conductive polymers, demonstrating excellent electrical conductivity.^[^ ^38^, ^39^, ^40^, ^41^ ^]^

#### 3.3.1 Homogeneous Rubber

The high aspect ratio of CNTs, while beneficial for forming conductive networks, also leads to significant challenges in achieving uniform dispersion within a rubber matrix. This is due to strong van der Waals forces between CNTs, causing them to readily entangle and agglomerate. To address the dispersion challenge, CNT/rubber composites are typically fabricated by solution mixing, with surface modification often used to improve filler–matrix compatibility. For example, Nina et al.^[^ ^42^ ^]^ prepared butyl rubber (BR)/CNT composites by solution mixing, achieving an EMI SE of 9–13 dB at 8 wt% loading. Similarly, Das et al.^[^ ^43^ ^]^ reported XNBR/CNT composites prepared by solution mixing, which reached an EMI SE of 27.4 dB at 15 phr.

Surface modification boosts CNTs dispersion in rubber by reducing clumping and improving compatibility. For example, Abraham et al.^[^ ^44^ ^]^ utilized ionic liquids as a modifier to functionalize CNTs through a grinding method, resulting in functionalized CNTs (f‐CNTs). Subsequently, they prepared styrene‐butadiene rubber (SBR)/f‐CNTs composite nanomaterials using a two‐roll shear mixing process. When the loading of f‐CNTs reached 10 phr, the composite achieved an EMI SE of 35.1 dB at 18 GHz. Daniel et al.^[^ ^45^ ^]^ loaded CNTs onto stearic acid‐modified layered double hydroxides (St‐LDH) and incorporated them into natural rubber (NR) to prepare NR/St‐LDH/CNT composites. The introduction of St‐LDH significantly enhanced the dispersion of CNTs. When the composite was loaded with 0.026 vol% CNTs, it achieved an EMI SE of 28 dB.

#### 3.3.2 Segregated Rubber

**Figure 2:**
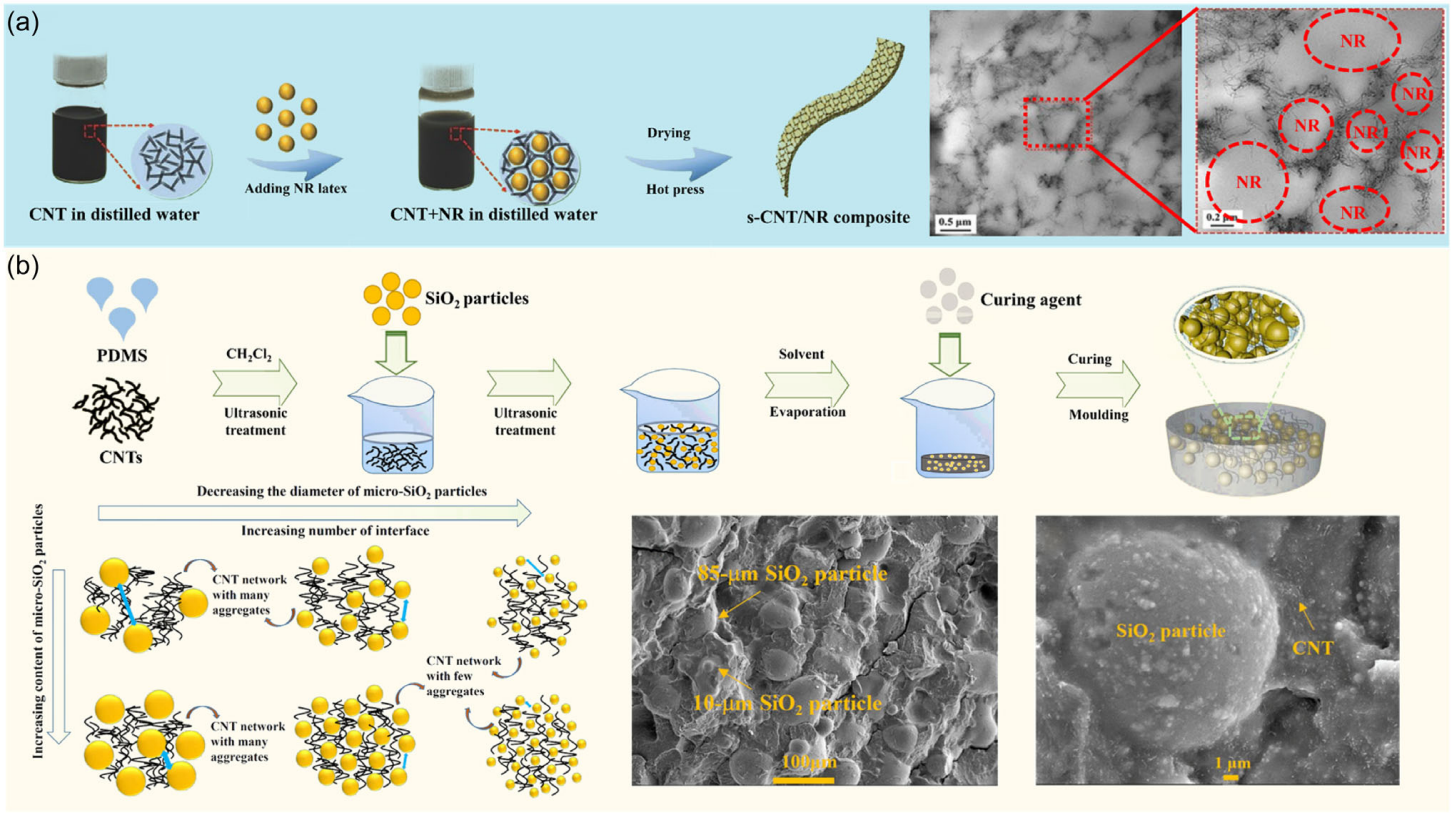
Fabrication schematics of a) NR‐latex repulsion‐induced segregated CNT/SR composites, and b) SiO_2_ exclusion‐induced segregated CNT/PDMS composites.^[^ ^47^, ^48^ ^]^

By controlling the spatial distribution of CNTs within the polymer matrix, such as by exploiting their selective distribution or matrix viscosity differences, CNTs can be enriched in specific regions of the rubber matrix, thereby constructing a 3D conductive network. For example, in the blend of NR and prepared polypropylene (PP), CNTs tend to disperse within the NR phase. When the loading of CNTs is 7 wt%, the composite achieves an SE of 29 dB at a frequency of 3 GHz.^[^ ^46^ ^]^ Additionally, NR latex particles can repel CNTs, causing them to cluster around the exterior of the latex particles and form a localized CNTs conductive network. As shown in **Figure** **2**a, a 600 μm thick composite film made from NR latex and CNTs (containing 5.0 wt% CNTs) exhibited an average EMI SE of 21.0 dB.^[^ ^47^ ^]^

Furthermore, incorporating nonconductive components like silica particles or fibers into the rubber matrix not only leverages the volume exclusion effect to facilitate the formation of a 3D conductive network by CNTs but also provides abundant interfaces to enhance electromagnetic wave loss. For example, by controlling the particle size of silica in PDMS, a composite with 3 vol% CNTs exhibited an EMI SE of 61.4 dB in the X‐band, as shown in Figure 2b.^[^ ^48^ ^]^ Similarly, the inclusion of 15 vol% cotton fiber in PDMS increased the EMI SE of a composite containing 3.0 vol% CNTs from ≈20 to ≈41 dB.^[^ ^49^ ^]^ The addition of silk fabric to NR latex enhanced the reflection of electromagnetic waves at the silk–NR and NR–NR interfaces, ultimately yielding an EMI SE of 19 dB at 300 MHz for a composite with 6 phr CNTs.^[^ ^50^ ^]^

Inspired by the volume occupancy effect, many researchers have employed dynamic vulcanization techniques to prepare conductive thermoplastic vulcanizate (TPV) composite or directly added cross‐linked rubber powder or recycled tire rubber powder into the rubber matrix. By utilizing the volume occupancy effect of the cross‐linked rubber phase, the distribution of CNTs can be effectively restricted.

**Figure 3:**
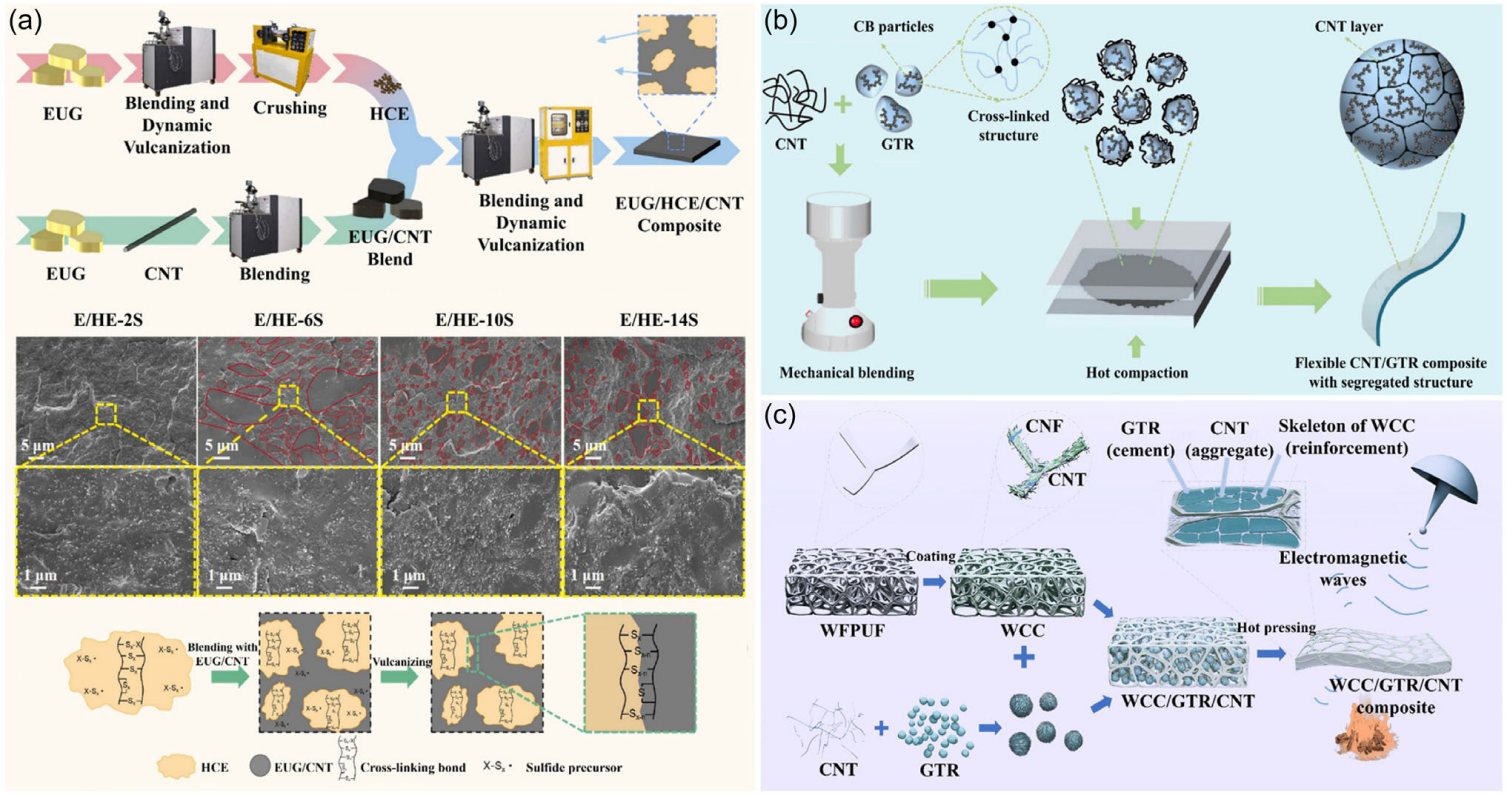
Fabrication schematics of a) vulcanized rubber powder backfilled segregated CNT/EUG composites, b) GTR hot‐pressed segregated CNT/GTR composites, and c) GTR backfilled CNT/GTR/PP composites.^[^ ^52^, ^53^, ^55^ ^]^

For example, in the PP/EPDM/CNT TPV composite, the vulcanized ethylene‐propylene‐diene rubber (EPDM) phase is used to limit the dispersion of CNTs. When the amount of CNTs is 4.1 vol%, the EMI SE of the composite reaches 29.8 dB in the X‐band, which is an improvement of 18.7% compared to the PP/EPDM/CNT thermoplastic elastomer composite that has not undergone dynamic vulcanization treatment.^[^ ^51^ ^]^ Han et al.^[^ ^52^ ^]^ reintroduced the prepared high‐cross‐linked Eucommia gum (EUG) powder into the EUG/CNT matrix. When the CNTs loading was 11.8 wt%, the EMI SE of the composite reached 83.0 dB, as shown in **Figure** **3**a. Jia et al.^[^ ^53^ ^]^ directly mixed tire rubber powder (GTR) with CNTs and prepared a typical phase‐separated CNT/GTR composite through hot pressing. The composite containing 5.0 wt% CNTs and a thickness of 2.6 mm achieved an EMI SE of up to 66.9 dB in the X‐band, as shown in Figure 3b. Moaref et al.^[^ ^54^ ^]^ mixed reclaimed rubber powder with CNTs and incorporated it into PP. The resulting composite, with a CNTs loading of only 5 vol% in a sample thickness of 2.5 mm, achieved an EMI SE of 30.4 dB, as shown in Figure 3c. Zhang et al.^[^ ^55^ ^]^ coated the surface of waste polyurethane foam (WFPUF) with a layer of CNT/cellulose nanofiber (CNF) composite material, forming a conductive foam known as WFPUF/CNT/CNF. Subsequently, GTR coated with CNTs was filled into the pores of the foam, resulting in an EMI SE of 53.8 dB for the 2 mm thick composite material.

#### 3.3.3 Multilayer Rubber

The multilayer architecture alternates conductive and insulating sheets, forcing incoming electromagnetic waves to undergo successive interface reflections and stepwise attenuation as they penetrate each conductive layer. Fabry–Pérot cavity resonances further amplify energy dissipation by trapping the waves between interfaces and promoting multiple‐beam interference. Concurrently, h‐BN provides an inherently high‐thermal‐conductivity phonon highway,^[^ ^56^ ^]^ while intercalated CNTs act as interlayer “thermal bridges.” These CNTs preserve the electromagnetic‐shielding network and simultaneously establish a 3D heat‐conduction path, endowing the composite with high electrical conductivity, high thermal conductivity, and efficient electromagnetic‐wave attenuation. For instance, multilayered conductive rubber composites, fabricated by alternately stacking NR/CNT conductive layers and NR/h‐BN insulating layers using latex mixing and vacuum filtration, exhibited an EMI SE of 31.4 dB and a thermal conductivity of 0.25 W m^−1^ K^−1^ when the CNTs content was ≈7.4 wt% and the h‐BN content was ≈10.7 wt%.^[^ ^57^ ^]^

#### 3.3.4 Foam Rubber

Beyond multilayer configurations, foam rubbers introduce porosity into the matrix, enabling lightweight structures with enhanced multiple scattering and high specific SE. Foam structures introduce a large number of pores, which not only reduce the material's density but also increase the reflection paths and scattering interfaces for electromagnetic waves. As a result, they offer unique advantages in terms of lightweight design and efficient shielding.^[^ ^48^ ^]^ Fan et al.^[^ ^58^ ^]^ employed supercritical carbon dioxide foaming to fabricate foam composites based on carboxyl‐terminated butadiene‐acrylonitrile copolymer (CTBN), epoxy resin (EP), and CNTs. When the CNTs loading was 5 wt%, the EMI SE of the composite material reached 22.9 dB in the frequency range of 12 to 18 GHz, and the specific SE (SSE) was 37.54 dB (g·cm^3^)^−1^. Tang et al.^[^ ^59^ ^]^ investigated the effect of pore structure on the shielding performance of conductive rubber foams and found that a graded porous structure exhibited both high EMI SE (52.7 dB) and absorption (A, 0.78), with a CNTs content of 8 wt%. Xie et al.^[^ ^60^, ^61^ ^]^ used expandable polymer microspheres as templates to prepare PDMS/CNT and NR/CNT foam composites. At a CNTs loading of 6.78 wt%, the PDMS/CNT composite exhibited an SSE of 74.18 dB cm^3^ g^−1^, along with an ultralow thermal conductivity of 0.028 W m^−1^ K^−1^. For the NR/CNT foam composite, the SSE reached 88.4 dB cm^3^ g^−1^, while the thermal conductivity was only 0.048 W m^−1^ K^−1^. Lu et al.^[^ ^62^ ^]^ fabricated 1.8 mm‐thick CNTs foams via chemical vapor deposition, achieving an EMI SE of 54.8 dB at X‐band and an SSE of 5480 dB cm^3^ g^−1^. After vacuum impregnation with PDMS, the composite material exhibited an EMI SE of 46.3 dB when the CNTs loading was below 1.0 wt%.

Overall, the different structural designs of CNT/rubber composites lead to distinct EMI shielding behaviors. Homogeneous systems suffer from limited CNT dispersion and weak filler‐matrix interactions, resulting in only moderate EMI SE at relatively high loadings. In contrast, segregated networks lower the percolation threshold and enhance interfacial polarization, multilayer configurations strengthen shielding through impedance matching and multiple reflections, and foam rubbers, with their porous architecture, maximize multiple scattering and deliver the highest specific SE despite their low density. As summarized in **Table** **2**, these structural designs clearly outperform conventional single‐component systems, which typically require much higher loadings to achieve sufficient EMI SE, thereby undermining flexibility and processability. However, despite their excellent performance, CNT‐ and graphene‐based composites still face higher production costs and challenges in large‐scale processing compared to CB, which constrains their direct industrial adoption. These limitations have driven the development of multicomponent synergistic systems to overcome the inherent drawbacks of single fillers.

**Table 2:** Comparison of electromagnetic shielding performance of different CNT/rubber composites.

Matrix	Filler loading of CNTs	Thickness [mm]	EMI SE [dB]	Frequency [GHz]	Refs.
BR	8 wt%	1	13	8–18	[42]
XNBR	15 phr	1	27.4	8–12	[43]
SBR	10 phr	5	35.1	2–18	[44]
NR	0.026 vol%	2	28	2–4	[45]
NR/PP	7 wt%	2	29	2–4	[46]
NR	5 wt%	0.6	21	8–12	[47]
PDMS	3 vol%	2	61.4	8–12	[48]
PDMS	3 vol%	1.2	41	8–12	[49]
NR	6 phr	2	19	0.3	[50]
EPDM/PP	4.1 vol%	1	29.8	8–12	[51]
EUG	11.8 wt%	2	83	8–12	[52]
GTR	5 wt%	2.6	66.9	8–12	[53]
EPDM/PP	5 vol%	2.5	30.4	8–12	[54]
GTR/WFPUF	6 wt%	2	53.8	8–12	[55]
NR	7.4 wt%	1.4	31.4	8–12	[57]
EPDM	7.8 wt%	3	35	8–12	[112]
CTBN/EP	5 wt%	2	22.9	12–18	[58]
SR	8 wt%	/	52.7	8–12	[59]
PDMS	6.78 wt%	2	44.5	8–12	[60]
NR	6.78 wt%	2	44.2	8–12	[61]
PDMS	1 wt%	2	46.3	8–12	[62]

## 4 Multicomponent Carbon Filler

Conductive rubber materials filled with single‐carbon fillers often suffer from poor filler dispersion, deteriorated processing properties at high loading levels, and a single electromagnetic wave attenuation mechanism. To address these issues, researchers have proposed a multicomponent filler composite strategy. By combining carbon fillers with other carbon materials, magnetic ferrites, or conductive fillers through composite hybridization or core–shell structural design, the synergistic effects between components can be fully utilized to significantly enhance electromagnetic shielding performance. Furthermore, by innovating the structure of composite materials, such as gradient frameworks, multilayer heterostructures, and foam architectures, a synergistic enhancement between the filler system and structural design can be achieved. This optimizes the multiple reflection‐absorption pathways of electromagnetic waves, ultimately leading to a leap in shielding efficiency.^[^ ^63^, ^64^, ^65^ ^]^ In particular, rubber matrices not only provide flexibility and lightweight characteristics but also allow processing into foams, multilayers, and stretchable structures. This tunability, which rigid polymer matrices generally lack, makes rubbers especially suitable for exploiting the synergistic effects of multicomponent filler systems.

### 4.1 Carbon–Carbon Filler Synergistic Systems

In multicomponent conductive filler systems, the combination of carbon‐based fillers has garnered significant attention due to their synergistic effects.^[^ ^66^, ^67^ ^]^ By combining different types of carbon fillers, the advantages of each can be fully utilized to construct more efficient conductive networks, thereby enhancing EMI shielding performance. Based on the distinct mechanisms underlying this synergy, carbon–carbon filler systems primarily operate through two key approaches: establishing mixed conductive networks and constructing special structures.

#### 4.1.1 Mixed Conductive Networks

The carbon–carbon hybrid system can effectively enhance the EMI SE of rubber by constructing a multiscale conductive network and increasing the electromagnetic wave reflection interfaces. For example, blending carbon fillers of different sizes can create conductive pathways that combine long‐range transport and bridging of aggregates.^[^ ^68^ ^]^ A typical example is the incorporation of CNTs and CB into isoprene rubber, where CNTs act as “transporters” to build long‐range conductive pathways and CB serves as a bridge to connect CNTs clusters, achieving an EMI SE of 30 dB for the composite material. In addition, hybrid carbon fillers can also effectively increase the electromagnetic wave reflection interfaces. For instance, by grinding to hybridize CNTs and GNP, and preparing CNTs‐GNP/EUG composites via solution mixing, an EMI SE of 42 dB was achieved in the X‐band.^[^ ^69^ ^]^ Similarly, by grinding to hybridize RGO and CNTs, and preparing RGO‐CNT/chloroprene rubber (CR) composites via mechanical blending, an EMI SE of ≈12 dB was obtained at a thickness of 0.5 mm.^[^ ^70^ ^]^

#### 4.1.2 Constructing Special Structures

Unlike conventional homogeneous blends, researchers have engineered multiscale ordered architectures—segregated, multilayer, foam, or aerogel structures—to precisely establish highly efficient conductive networks and multiple reflection interfaces, markedly enhancing electromagnetic wave reflection and dissipation.^[^ ^71^, ^72^ ^]^

**Figure 4:**
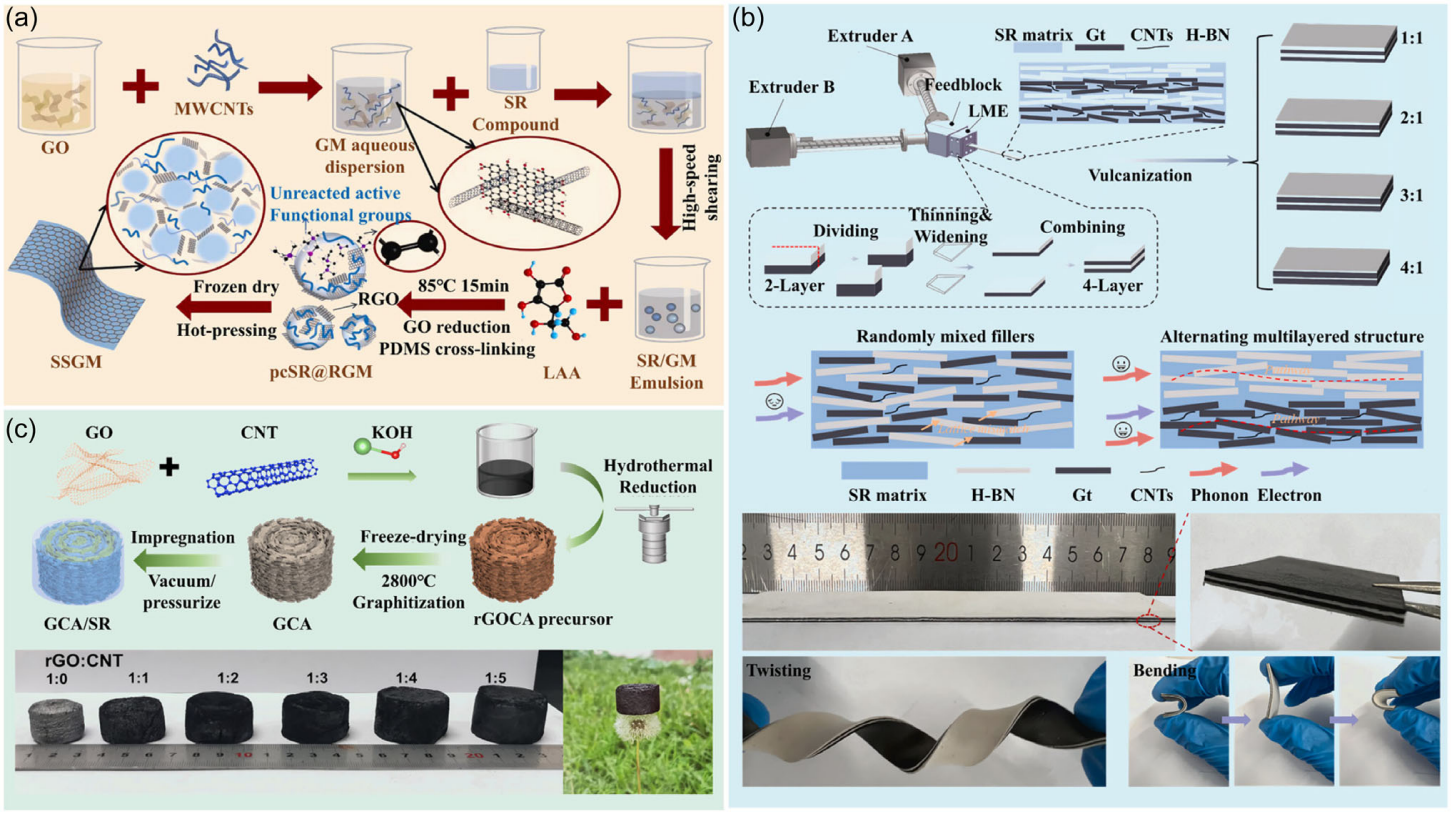
Fabrication schematics of a) pickering‐emulsion‐coated segregated SR@RGO/CNT composites, b) coextruded alternating multilayer Gt/CNT/h‐BN/SR composites, and c) 3D graphene/CNT aerogels/SR composites.^[^ ^73^, ^74^, ^76^ ^]^

Among them, Wang et al.^[^ ^73^ ^]^ utilized the pickering emulsion method to hybridize RGO and CNTs as fillers with precrosslinked SR particles, successfully preparing core–shell structured SR@filler particles. By precisely controlling the distribution of the fillers on the particle surface, they constructed a segregated conductive network, ultimately achieving an EMI SE of 41.4 dB (**Figure** **4**a). Another multilayer strategy was proposed by Ji et al.^[^ ^74^ ^]^ Using coextrusion technology, they achieved an alternating distribution of graphite (Gt), CNTs, and h‐BN to form alternating multilayer SR composites. This unique design significantly enhanced EMI shielding performance through multiple reflections of electromagnetic waves, resulting in an EMI SE of 31–43.2 dB (Figure 4b). For lightweight design, Jeddi et al.^[^ ^75^ ^]^ coated polyurethane foam with CB/GNP, utilizing its structure for EM wave reflection and refraction. At a thickness of 2 mm, they achieved an EMI SE of over 30 dB. Liu et al.^[^ ^76^ ^]^ fabricated anisotropic 3D graphene/CNT aerogels through a KOH‐induced hydrothermal reaction and subsequent graphitization at 2800 °C. This 3D network structure not only improved the electrical conductivity but also enhanced the EMI shielding performance, achieving a maximum electrical conductivity and EMI SE of 42 dB in the K‐band, as shown in Figure 4c.

In summary, carbon–carbon hybrids construct multiscale conductive networks and interface‐rich architectures, where ohmic conduction, interfacial polarization, and multiple scattering jointly enhance EMI attenuation. From an engineering perspective, they are lightweight and corrosion‐resistant, suitable for ordinary EMI protection, but dispersion of nanocarbon fillers remains a critical challenge and the overall SE is relatively moderate.

### 4.2 Carbon–Metal Filler Synergistic System

Compared to carbon materials, metals generally possess higher electrical conductivity, enabling them to respond rapidly to electromagnetic fields through free electrons, resulting in significant reflection loss. Therefore, combining carbon materials with metal fillers holds the promise of significantly enhancing electromagnetic shielding performance while reducing material cost and density. In particular, coating carbon fillers with a metal layer via techniques such as electroless plating can not only improve the material's conductivity but also fully leverage the reflection loss advantages of metals.^[^ ^77^ ^]^

A 3D bicontinuous RGO/silver nanowire (AgNW) conductive framework, fabricated via sol‐gel method, was infiltrated with PDMS to create PDMS/RGO/AgNW composites.^[^ ^78^ ^]^ This interconnected 3D structure enhances electron transport pathways, significantly boosting electrical conductivity. With 0.43 wt% RGO and 0.33 wt% AgNW loading, the composite achieved a maximum EMI SE of 34.1 dB. In a separate approach, electroless plated nickel‐coated carbon fibers (Ni@CF) were incorporated into an SR matrix. A 0.7 mm thick Ni@CF/SR composite containing 60 wt% filler exhibited an EMI SE of 42.6 dB.^[^ ^79^ ^]^ Similarly, NR/Ag@CF composites combining silver‐electroless‐plated carbon fibers with NR leveraged silver's conductivity and CF's aspect ratio. At 45 phr Ag@CF loading, these composites yielded an EMI SE of 111 dB, demonstrating the effectiveness of integrated carbon–metal properties.^[^ ^80^ ^]^

Taken together, carbon–metal systems rely on the high conductivity of metals to induce strong surface reflection, while carbon networks provide additional conductive pathways and interfacial polarization, with multilayer or porous designs introducing multiple reflections to further improve EMI shielding at reduced thickness. Practically, they deliver high shielding efficiency, but increased weight, processing complexity, and possible corrosion of metallic components limit their applicability in lightweight and long‐term stable devices.

### 4.3 Carbon–Magnetic Filler Synergistic System

Magnetic fillers mainly convert electromagnetic waves into thermal energy dissipation through magnetic loss and ferromagnetic resonance mechanisms.^[^ ^81^, ^82^, ^83^ ^]^ By using magnetic fillers in combination with conductive fillers, or by preparing multilayer structures, additional electromagnetic wave absorption mechanisms can be introduced, or a triple mechanism of absorption–reflection–reabsorption can be constructed. This not only enhances the shielding effect but also reduces the secondary pollution of electromagnetic waves.

#### 4.3.1 Heterogeneous Interface

By combining conductive fillers with magnetic fillers to construct a carbon–magnetic hybrid system, a large number of heterogeneous interfaces can be formed within the rubber matrix. In this system, the conductive fillers interconnect to form a continuous 3D conductive network. The magnetic fillers play a dual role: they not only supplement the conductive network to enhance dielectric loss but also directly dissipate electromagnetic wave energy through the magnetic loss mechanism. In particular, at the carbon–magnetic heterogeneous interface, the electromagnetic waves undergo multiple reflections and refractions before being efficiently absorbed by the magnetic components and converted into thermal energy, thereby significantly enhancing the overall electromagnetic wave attenuation capability.^[^ ^84^, ^85^, ^86^, ^87^, ^88^, ^89^, ^90^, ^91^ ^]^

**Figure 5:**
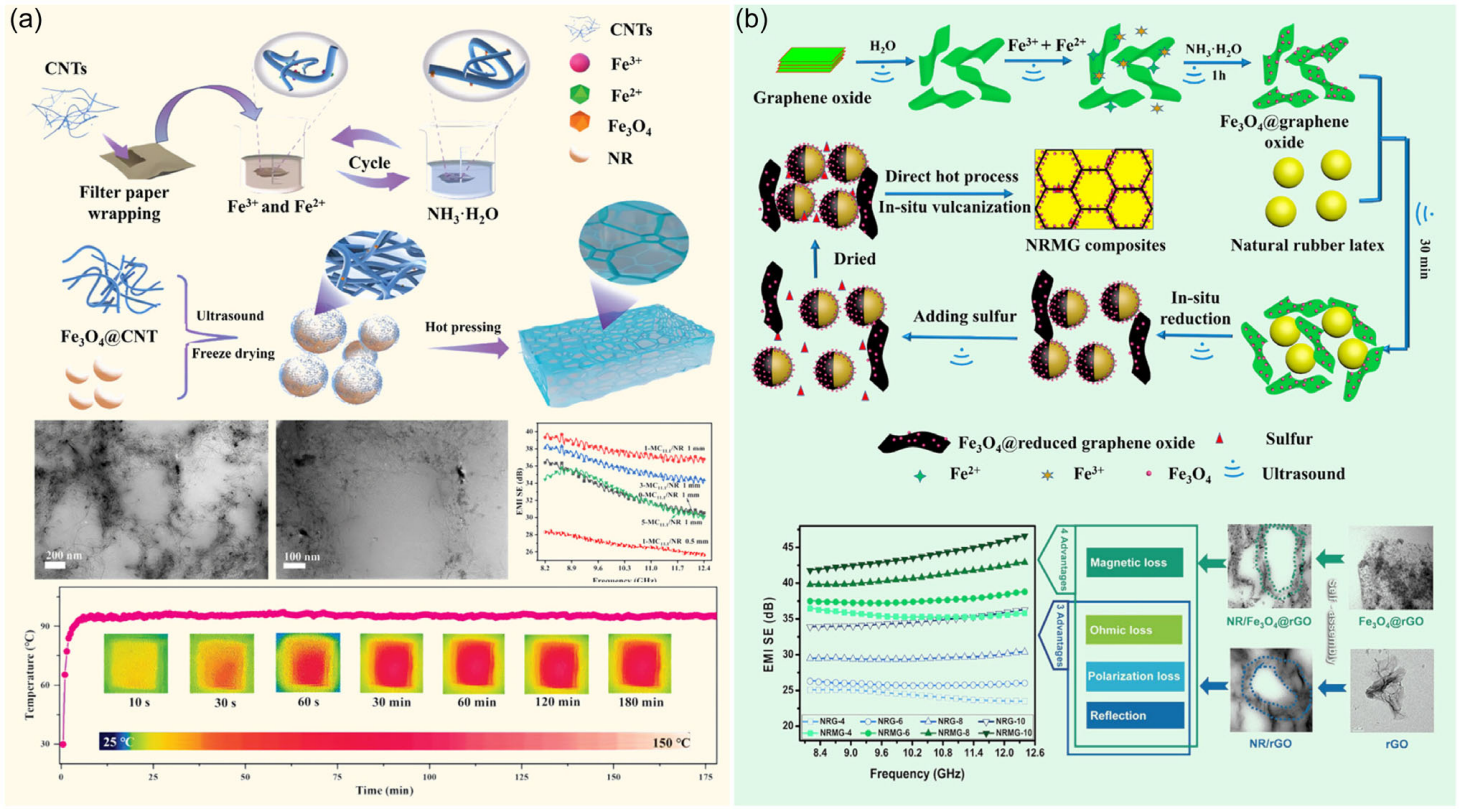
Fabrication schematics of segregated rubber composites via emulsion mixing: a) Fe_3_O_4_@CNTs/NR composites and b) Fe_3_O_4_@RGO/NR composites.^[^ ^93^, ^94^ ^]^

For example, Zhang et al.^[^ ^92^ ^]^ exploiting the incompatibility of NR and NBR to create an NR/NBR/CNT/Fe_3_O_4_ composite with numerous heterogeneous interfaces yielded an EMI SE of 29.4 dB. To optimize the construction of heterogeneous interfaces, Fe_3_O_4_@CNTs core–shell structures were synthesized using the coprecipitation method and then NR/Fe_3_O_4_@CNTs composite materials with structural isolation were prepared via a latex blending process.^[^ ^93^ ^]^ When the filler content was 11.1 phr, the 0.5 mm thick sample achieved a total SE of 26.6 dB in the X‐band, with a reflection coefficient (*R*) as low as 0.24 (**Figure** **5**a). Similarly, by constructing the Fe_3_O_4_@RGO/NR composite material (Figure 5b), its SE was enhanced by 41% compared to the NR/RGO system with the same RGO content (30 dB vs. 42.4 dB), which verified the synergistic advantages of the magnetic components and the heterogeneous interfaces.^[^ ^94^, ^95^ ^]^ After undergoing complex cyclic deformation, the shielding attenuation rate of this material was ≤2.9%, while the performance of the pure NR/RGO system degraded by 16% under extreme working conditions.

#### 4.3.2 Filler Orientation

By precisely tailoring the spatial arrangement of fillers within the matrix, the electromagnetic‐shielding performance of composites can be markedly enhanced. Strategies such as prearranging the filler skeleton followed by rubber infiltration or magnetically directing the assembly of magnetic particles both create anisotropic conductive networks. This ordered architecture not only optimizes the conductive pathways but also introduces a hierarchical interfacial structure that forces electromagnetic waves to undergo multiple, well‐ordered reflections and refractions inside the material. Consequently, higher electromagnetic dissipation efficiency is achieved at the same filler loading.

Xu et al.^[^ ^96^ ^]^ found through comparative studies that when magnetic cobalt‐rich microwires (M) and graphene fibers (G) were periodically arranged in PDMS, even at a filler content of 0.059 wt%, an EMI SE of 18 dB could be achieved, which is much higher than that of the randomly distributed state (6 dB). Guo et al.^[^ ^97^ ^]^ and Xie et al.^[^ ^98^ ^]^ used magnetic field induction to direct the alignment of fillers within the rubber matrix. For example, Guo et al.'s study found that under a magnetic field of 180 mT, the Fe_3_O_4_@RGO/SR composite material exhibited the most significant oriented alignment, with an EMI SE reaching ≈17.8 dB, whereas without the application of a magnetic field, its EMI SE was significantly reduced (12–14 dB).

#### 4.3.3 Multilayer Structure

Multilayer structural design serves as a critical strategy for breakthrough advancements in electromagnetic shielding performance. This approach extends the transmission path of electromagnetic waves within the material, inducing multiple reflections at heterogeneous interfaces. Simultaneously, through spatial distribution control of magnetic and conductive components, it establishes a directed “absorption–reflection–reabsorption” cyclic attenuation mechanism. This coupling effect harnesses the dielectric/magnetic losses of magnetic layers to absorb incident wave energy while utilizing the high reflectivity of conductive layers to redirect residual electromagnetic waves back to the absorption layer (AL) for secondary dissipation. Thereby, it synergistically enhances the overall electromagnetic wave attenuation efficiency.

**Figure 6:**
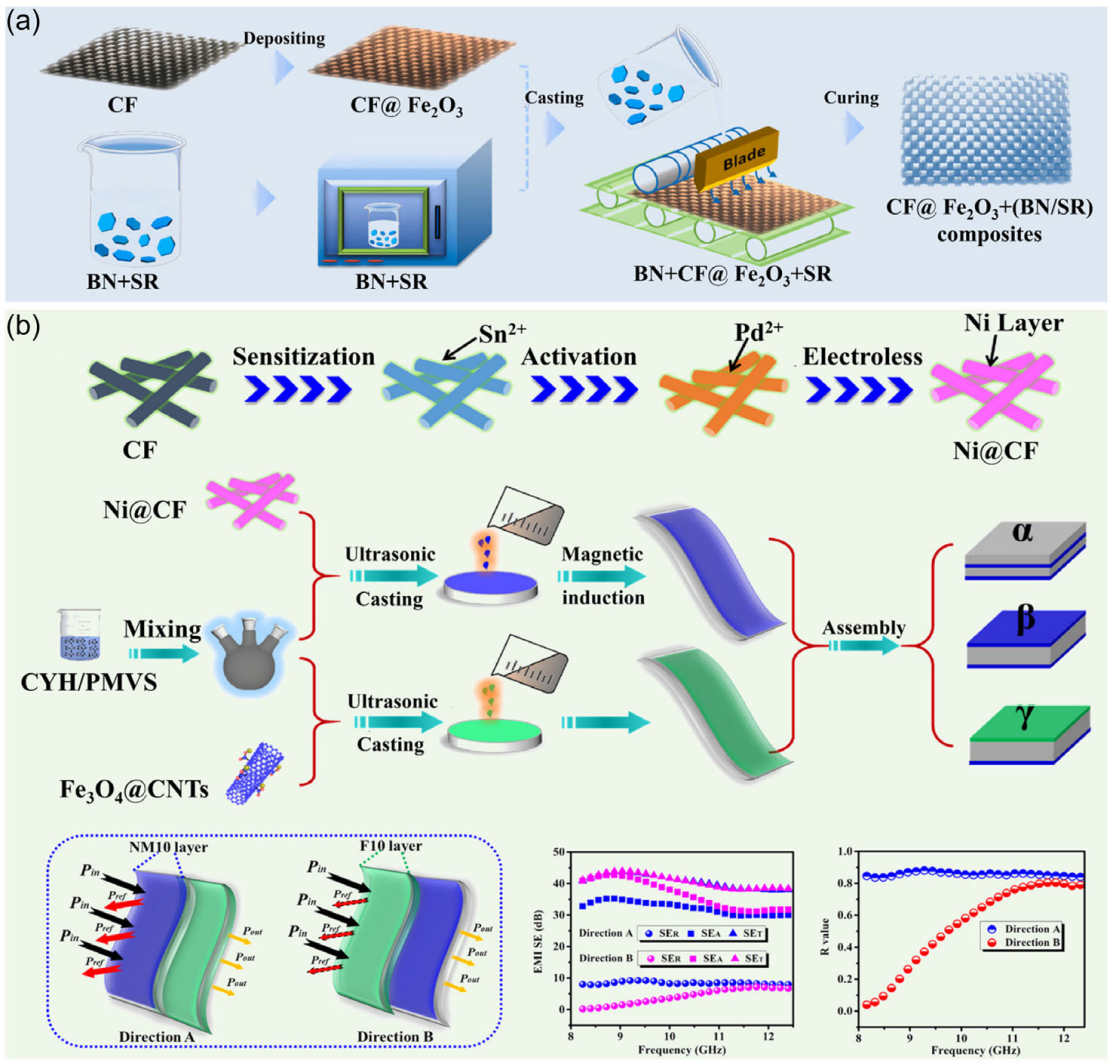
Fabrication schematics of a) multilayer h‐BN/SR‐CFF@Fe_2_O_3_ composites and b) multilayer Ni@CF/SR‐Fe_3_O_4_@CNTs/SR composites.^[^ ^99^, ^103^ ^]^

For example, when an h‐BN/SR outer layer is combined with a Fe_2_O_3_‐modified carbon‐fiber middle layer (CFF@Fe_2_O_3_), the synergy between 20.6 wt% h‐BN and 45.5 wt% CFF@Fe_2_O_3_ simultaneously delivers a thermal conductivity of 3.86 W m^−1^ K^−1^ and an EMI SE of 37.7 dB, as shown in **Figure** **6**a.^[^ ^99^ ^]^ Likewise, the cooperation between an SR/CNT outer layer and a Ni‐Fe/carbon–fiber cloth middle layer endows the composite with an EMI SE of 60.2–85.5 dB across 0–4800 MHz.^[^ ^100^ ^]^ Li et al.^[^ ^101^ ^]^ assembled a porous Fe_3_O_4_@CNTs AL on top of a silver‐plated nonwoven fabric (Ag@NWF) reflection layer (RL); with the RL only 0.18 mm thick, the material exhibits an EMI SE of 34.4 dB and an ultralow reflection coefficient of 0.1. Utilizing the same AL/RL design with porous Fe_3_O_4_@CNTs over Ag@NWF, Yang et al.^[^ ^102^ ^]^ achieved 88.4 dB EMI SE. This performance was attained with only 2.08 vol% Fe_3_O_4_@CNTs and 0.81 vol% Ag loading, a density of 0.38 g cm^−3^, and a reflection coefficient of 0.54. Furthermore, by magnetically inducing a graded enrichment of Ni@CF in SR and layer‐by‐layer stacking with SR/Fe_3_O_4_@CNTs (Figure 6b), they produced a 1.4 mm thick composite that reaches 60.8 dB EMI SE at 8.2 GHz with a 96% electromagnetic‐wave absorption efficiency.^[^ ^103^ ^]^

#### 4.3.4 Foam Structure

Foam structures leverage their distinctive lightweight and porous characteristics to significantly reduce material density while providing complex 3D transmission pathways for electromagnetic waves. The interconnected pore networks within these structures induce multilevel scattering and interfacial reflections of electromagnetic waves, prolonging their dwell time within the material. Furthermore, the multiheterostructures formed at solid–gas interfaces enhance the impedance matching of incident waves while synergizing with the intrinsic dielectric/magnetic loss mechanisms of the material to facilitate efficient electromagnetic energy dissipation. This coupling between physical structural regulation and functional component properties establishes foam architectures as promising candidates for lightweight and high‐efficiency EMI shielding.^[^ ^104^, ^105^, ^106^, ^107^ ^]^

**Figure 7:**
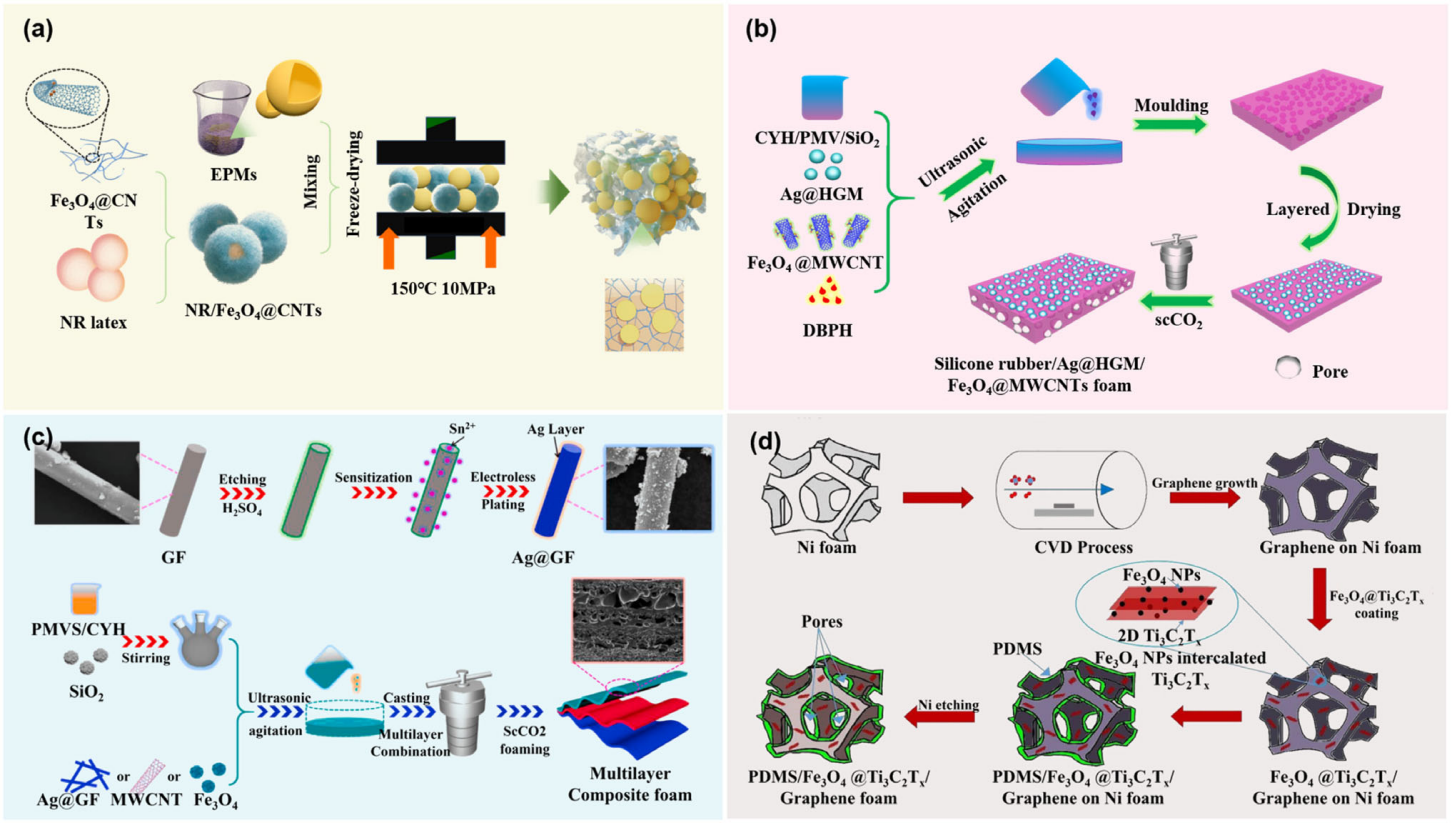
Fabrication schematics of a) NR/Fe_3_O_4_@CNTs foams composite, b) SR/Ag@HGMs/ Fe_3_O_4_@CNTs foam composite, c) SR/Ag@GF/Fe_3_O_4_/CNT foam composite, and d) PDMS/Fe_3_O_4_@Ti_3_C_2_T_X_/Graphene foam composite.^[^ ^108^, ^109^, ^110^, ^111^ ^]^

Similarly, Wei et al.^[^ ^108^ ^]^ utilized a template foaming method to prepare NR/Fe_3_O_4_@CNTs foam. With the addition of only 4.6 wt% Fe_3_O_4_@CNTs, it achieved an EMI SE of 23.2 dB and an extremely low reflection coefficient (0.19), as shown in **Figure** **7**a. Yang et al.^[^ ^109^ ^]^ introduced Fe_3_O_4_@CNTs nanoparticles into the gradient structure of an SR/Ag‐coated hollow glass microsphere composite material (Figure 7b). This ingenious structural design not only reduced the material density but also optimized the propagation path of electromagnetic waves. Ultimately, the composite foam achieved an average EMI SE of 59.4 dB and a low reflection coefficient of 0.59. They team also prepared silver‐plated glass fibers (Ag@GF) via chemical plating.^[^ ^110^ ^]^ By combining solution mixing and layer‐by‐layer assembly, they developed a gradient porous SR composite foam (Figure 7c), which realized a high average EMI SE of 78.6 dB and an absorption coefficient (*A*) of 0.82. Nguyen et al. hybridized Fe_3_O_4_ nanoparticles into the interlayers of Ti_3_C_2_T_X_ and coated this hybrid onto the surface of graphene aerogels, followed by encapsulation with PDMS, as shown in Figure 7d. The composite material containing 11.53 wt% Fe_3_O_4_@Ti_3_C_2_T_X_ achieved average EMI SE values of 80 dB in the X‐band and 77 dB in the Ka‐band at a thickness of 1 mm.^[^ ^111^ ^]^

Overall, carbon–magnetic systems combine conductive network reflection and dissipation with magnetic dipole resonance and interfacial polarization, thereby extending attenuation paths and increasing absorption contribution to EMI shielding. From an application viewpoint, they significantly enhance absorption and are attractive for low‐reflection requirements, but the higher density and reduced elasticity introduced by magnetic fillers may hinder their use in ultralightweight flexible devices.

Collectively, multicomponent systems can combine the lightweight advantage of carbon fillers, the strong reflection of metals, and the additional loss channels of magnetic components, while structural designs such as multilayers and foams can further amplify these effects. By tuning filler composition and structural design, EMI shielding rubbers can be tailored to meet diverse practical requirements in terms of shielding performance, mechanical properties, weight, and corrosion resistance.

These structural strategies derive additional advantages from the intrinsic flexibility and elasticity of rubber matrices, which allow conductive pathways to be maintained or reconstructed under deformation. This unique property links filler distribution, pore morphology, and orientation directly to EMI shielding performance, further distinguishing rubber‐based composites from rigid matrices. For clarity, **Table** **3** provides a concise comparison of these synergistic strategies, complementing the detailed discussion above. Complementing Table 3's summary of synergistic strategies, **Table** **4** compares the resulting EMI shielding performance across representative multicomponent carbon–filler/rubber composites.

**Table 3:** Summary of synergistic strategies in conductive rubber composites.

Type	Strategy	Key features/benefits
Filler–filler synergy	Carbon–carbon system	Lightweight, corrosion‐resistant; generally moderate SE
	Carbon–metal system	High shielding efficiency; risk of added weight and corrosion
	Carbon–magnetic system	Additional magnetic loss; reduced secondary reflection
Filler–structure synergy	Homogeneous structure	Simple processing
	Oriented structure	Anisotropic EMI shielding
	Multilayer structure	Impedance matching and multiple reflections; tunable performance via layer programming
	Foam structure	Lightweight, porous; multiple scattering yields high specific SE
	Heterogeneous interface	Strong interfacial polarization

**Table 4:** Comparison of electromagnetic shielding performance of different multicomponent carbon–filler/rubber composites.

Matrix	Filler loading	Thickness [mm]	EMI SE [dB]	Frequency [GHz]	Refs.
NR	20 wt%CB + 20 wt%CNTs	1	30	12–18	[68]
CR	0.9 phr RGO + 4 phr CNTs	0.5	11.9	3–3.5	[69]
EUG	9.62 wt%CNTs + 3.85 wt%GNP	2	42.5	8–12	[70]
NR	5 wt%CNTs + 50 wt%MXene	0.2	49.4	8–12	[71]
SR	1 wt%CB + CFP@MnO_2_	1	32.2	8–12	[72]
SR	4.5 wt%RGO + 3.2 wt%CNTs	2	41.4	8–12	[73]
SR	54.3 wt%Gt + 0.45 wt%CNTs	1.5	43.2	8–12	[74]
RTV	3.16 wt%GNP + 12.63 wt%CB	2	30	8–12	[75]
SR	2.77 wt%RGO/CNTs	2	42	18–27	[76]
PDMS	0.43 wt%RGO + 0.33 wt%AgNWs	2	34.1	8–12	[78]
SR	60 wt% Ni@CF	0.7	42.6	8–12	[79]
NR	45 phr Ag@CF	/	111	8–12	[80]
NR	6 phr GNP + 6 phr CoNWs	3	38	3–18	[84]
SR	5 wt%CNTs + 10 wt%Red Mud	2	83.4	8–12	[85]
EUG/EP	5 wt%CB + 25 wt%MZF	2	30.9	8–12	[86]
NR/NBR	12 wt%CNTs + 12 wt%Fe_3_O_4_	2	29.4	8–12	[92]
NR	11.1 phr Fe_3_O_4_@CNTs	0.5	26.6	8–12	[93]
NR	10 phr Fe_3_O_4_@RGO	1.8	42.4	8–12	[94]
PDMS	0.059 wt% graphene fibers and cobalt‐rich microwires	2	18	8–12	[96]
SR	25 wt% Fe_3_O_4_@RGO	2	17.8	8–12	[97]
SR	25 wt% Fe_3_O_4_@RGO	2	21.6	8–12	[98]
SR	20.6 wt% h‐BN + 45.5 wt% Fe_2_O_3_@CF	0.74	50.9	8–12	[99]
SR	10 wt%CNTs + CF@Ni‐Fe	1	85.5	0–4.8	[100]
NR	50 wt%CNTs + 3v F@rLG	2	34.4	128–1218	[101]
SR	2.08 vol% Fe_3_O_4_@CNTs + 0.81 vol% Ag	2	88.4	8–12	[102]
SR	10 wt% Fe_3_O_4_@CNTs	1.4	60.8	8–12	[103]
SR	10 wt%GNP + 9 wt%NiFe_2_O_4_	3	36.7	8–12	[104]
SR	10 wt%CNTs + 20 wt%Fe_3_O_4_	2	27.5	8–12	[105]
PDMS	12 wt% GF/h‐Fe_3_O_4_	2	70.4	8–12	[106]
NR	4.6 wt% Fe_3_O_4_@CNTs	2	22.3	8–12	[108]
SR	0.51 vol% Ag + 1.6 vol%Fe_3_O_4_@CNTs	2	59.4	8–12	[109]
SR	10.7 wt% Ag + 20 wt% Fe_3_O_4_ + (3 wt%,2 wt%,1 wt%) CNTs	/	78.6	28–1218	[110]
PDMS	11.53 wt% Fe_3_O_4_@Ti_3_C_2_T_X_	1	80	8–12	[111]

## 5 Conclusion and Future Outlooks

**Figure 8:**
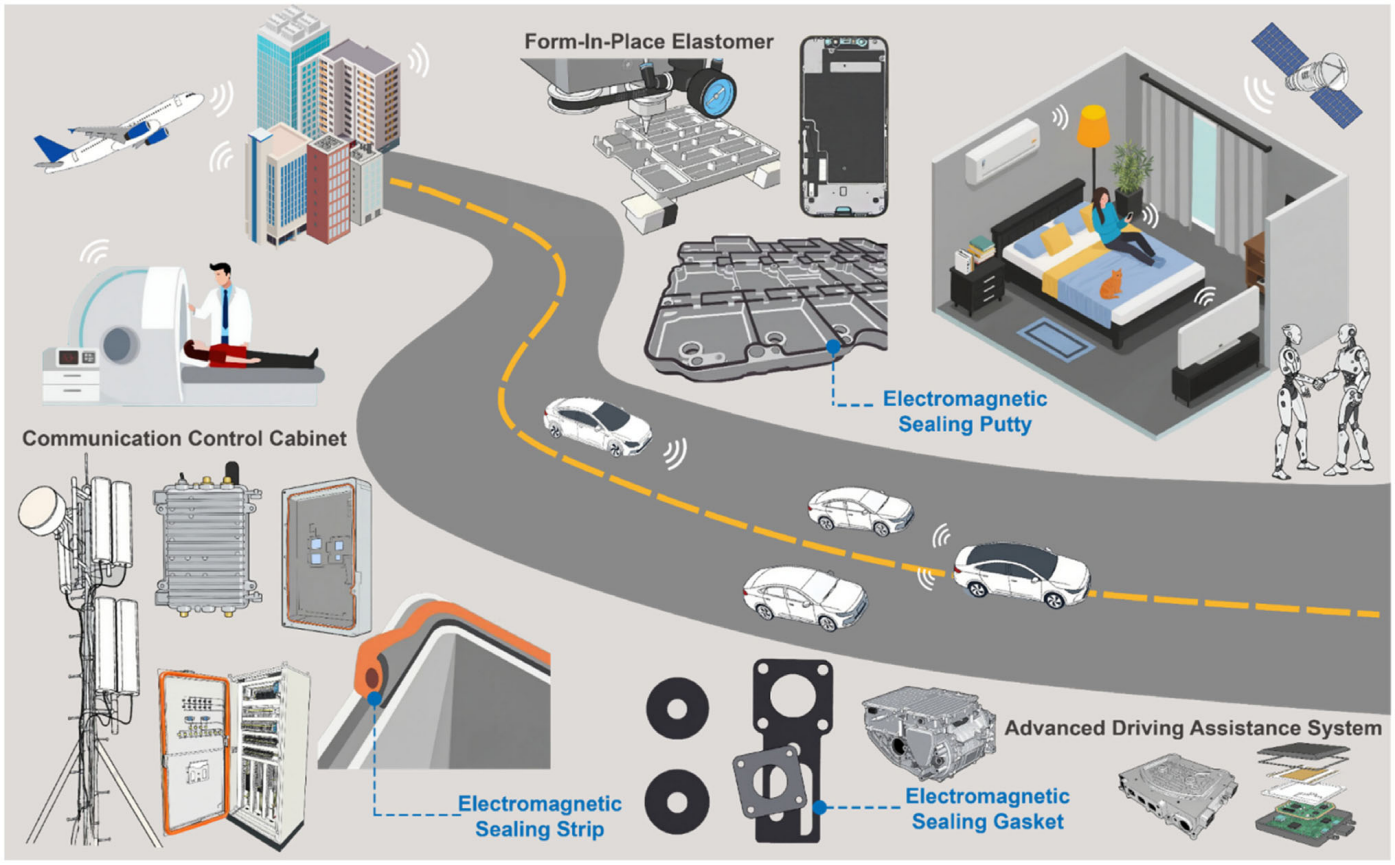
The application scenarios and forms of electromagnetic shielding rubber.

As illustrated in **Figure** **8**, conductive rubber has been widely applied in communication systems, vehicles, and consumer electronics in the form of sealing strips, gaskets, and putties. These applications demonstrate its broad potential, but they also place increasing demands on performance to cope with complex electromagnetic environments. Overall, carbon–carbon systems are lightweight and corrosion‐resistant but generally provide only moderate SE. Carbon–metal systems achieve high SE at reduced thickness but increase weight, processing complexity, and susceptibility to corrosion. Carbon–magnetic systems enhance absorption and reduce reflection, yet they raise density and may compromise elasticity.

Despite these advances, critical bottlenecks remain, including high filler loading requirements and difficulties in achieving uniform dispersion and stable conductive networks, as well as insufficient evaluation of interfacial adhesion and long‐term reliability under coupled service conditions. In view of these challenges, future research should focus on the following points:

### 5.1 Service Reliability and Degradation Mechanisms

Future research needs to delve into the long‐term performance evolution and degradation mechanisms of conductive rubber under complex service conditions (such as large deformation, cyclic loading, long‐term compression, environmental ageing, and exposure to corrosive media) and under the coupling of multiple physical fields (e.g., mechanical, thermal, and chemical). The focus should be on revealing the underlying failure mechanisms (including fatigue crack initiation/propagation, interfacial debonding, electrochemical corrosion at heterogeneous interfaces, and irreversible damage to the conductive network), establishing predictive models for service life, and developing strategies for enhancing long‐term reliability and durability.

### 5.2 Integrated Structural–Functional Design

Carbon‐based conductive rubber still faces performance bottlenecks in terms of electromagnetic shielding and mechanical properties. High filler loading often makes it difficult to balance the two. Future research could draw on the design concepts of metal mesh skeleton/rubber composite materials, leveraging synergistic effects to explore 3D interpenetrating network structures, bionic design, and other approaches to develop lightweight, high‐performance composite materials. Key research directions include the mechanisms of interfacial stress transfer, the adhesion mechanisms at the rubber–skeleton interface, and the design of high‐strength, high‐toughness, high‐conductivity, and fatigue‐resistant adhesion at heterogeneous material interfaces under dynamic service conditions.

### 5.3 Intelligent Responsive Materials

Fully utilizing the programable reconstruction characteristics of conductive networks triggered by the large deformation of rubber, research should be conducted on the microscopic topological evolution and electromagnetic parameter response patterns under nonequilibrium states of rubber (such as stretching, compression, and twisting). Based on this, intelligent electromagnetic responsive switches should be designed and developed, such as precise switching between shielding and transmission states. These breakthroughs will directly promote disruptive engineering designs like adaptive electromagnetic skins and mechanically tunable communication filters.

For example, incorporating microencapsulated phase‐change materials into conductive rubber can achieve thermally induced fine‐tuning of EMI SE by altering dielectric relaxation and contact resistance during the phase transition, while conductive rubber foams can realize a pronounced “on‐off” switching behavior of electromagnetic shielding under mechanical compression.

### 5.4 Multifunctionality and Sustainability

Beyond shielding efficiency, future research should integrate multifunctionality into conductive rubber composites. Segregated and multilayer structures can form conductive‐thermal networks for combined thermal management and EMI shielding, while foamed rubbers with designed open‐ or closed‐cell structures can add thermal insulation or acoustic absorption. Conductive thermoplastic vulcanizates combine elasticity with melt reprocessability, making recyclable shielding materials possible. Self‐healing can be achieved through dynamic covalent bonds, Zn^2+^‐carboxylate coordination, or liquid metals that restore conductive pathways after damage. At the same time, scalable processes such as foaming, lamination, and dynamic vulcanization, together with recyclable rubbers and fillers, will be essential to ensure sustainable and industrially viable solutions.

## Conflict of Interest

The authors declare no conflict of interest.
